# Commencement Exercises of the Southern Medical College—Session of 1885-86

**Published:** 1886-03-20

**Authors:** 


### COMMENCEMENT EXERCISES OF THE SOUTHERN MEDICAL COLLEGE—SESSION OF 1885–'86.

The Commencement Exercises of the Southern Medical College—Session 1885–'86—took place at the Opera House, Atlanta, February 25, 1886.

Notwithstanding it was a rainy and inclement evening, there was a laige audience present, including many ladies, whose interest in the occasion had brought them out in the face of the unpleasant weather.

Upon raising the curtain, at 8 o'clook, there was presented an imposing array of medical graduates on either side, and in the center Rev. J. B. Hawthorne, the orator, with Professors Gaston, Powell, Word, Bizzell, Ray, Nicolson, Hobbs, and Burns, with Rev. Dr. Eddy, Hon. Mr. Fite, Colonel Kiser, and other prominent gentlemen.

The exercises were opened by an eloquent and appropriate prayer by Rev. Dr. Eddy ; after which each student, at the call of his name, approached, and, with a polite bow, received his diploma at the hands of the Dean. Then, as they stood in two divisions, one on either side of the stage, the degree of Doctor of Medicine was conferred in official and appropriate terms by Professor Powell, president of the Board and Faculty, who also addressed them in the following words, characterized by his usual ability, and designed to instruct them in science, morals and religion :

GENTLEMEN :

As the brook, whispering and laughing in its glee, glides out from the shadows of woodland bowers, and pours its crystal bright waters into the river's broad bosom, to flow on with that mighty stream to the boundless ocean, so you, my dear young brethren, have just emerged from under the teachings of the Southern Medical College into a wider domain—the duties and responsibilities of the true Physician and the legitimate Doctor of Medicine.

In performing this, the last duty from Instructor to Student, let me remind you that the noble profession you have chosen is of Divine origin, and you, as its representatives, should have a profound conception ot its sacred mission in your hands.

At its earliest birth, the genius of Medicine received the baptism of Divine approval ; and as the great Giver of all Good had from the beginning created remedial agents for the healing of the nations, so this benediction rested upon the earliest efforts of the honest and conscientious physician to discover and adapt beneficent and effectual remedies that would lessen the physical ills to which the human race became doomed after the fall of man.

Our brethren of the Masonic fraternity feel much pride in dating their noble organization as far back in the ages as the reign of King Solomon, its illustrious founder ; but the reign of Medicine is of much greater antiquity, as shown in the pages of the Holy Scriptures. Medical scientists of modern times have been amazed at the wonderful knowledge of Moses, exhibited in hygeinic art, and who established sanitary rules in those ancient days that would be well for us to follow in this progressive nineteenth century. It is said that the Levites, who constituted an hereditary nobility among the Hebrews, were both the doctors and the judges of the people; and Solomon, the wisest of men, wiote on the cure of diseases by natural means. In the Old Testament, the physician, whenever mentioned, is spoken of with great deference, and, sometimes, even with reverence.

From the rude art of healing among the Egyptians, to the superstitious idealism of the Greeks and Romans, the humane and noble object of medicine was to discover truths supported by facts, whose application to the end in view would enable the Healing Art to combat diseases, and, though it often failed, as in these latter days, yet its purpose was respected. In this quest, the medical investigator, in the classic ages, was often hindered by a false phylosophy, which erected imaginary laws to account for observable phenomena, and endeavored to make the operations of Nature accord with their theories, instead of having the latter in harmony with Nature's laws, which are never inexact. Neither Melampus nor Galen observed this strong point in medical research ; but still their labors in the search of Truth, were so many steps towards the beginning of that advance movement in Medicine which has enabled it to reach its high position at the present day.

In what is called the Dark Ages of History, the medical, as well as the scholastic world, was shrouded in the gloom of that period, from which it did not wholly emerge until Mondini, the anatomist of Bologna, drew aside the veil and revealed the coming dawn of modern medicine.

In France, surgery now took great progressive steps, and the whole medical world received an impetus that has steadily pressed onward through the succeeding years. As the grand procession moves forward, we distinguish among its illustrious figures Francis Bacon, the "father of positive science" ; Gilbert, the discoverer of terrestrial magnetism ; Francis Redi, who overthrew the doctrine of spontaneous generation ; and the great Harvey, Lower, Boyle, Hook, and Mayow. Then follow the philanthrhopist Bœrhave ; William Cullen, who so successfully combatted false facts ;

John Hunter, and Astley Cooper; while bringing up the rear in the roll of honor William Farr, Edward Parkins, and John Simon, illustrious pioneers of hygienic art, move forward with the uplifted banner of Modern Sanitary Science.

If time would permit, I could mention many American names, famous in medical science and practice, and though they have passed from the troubles of active life to rest in the Spirit Land, these names are all carved on the temple of Fame, and are enshrined in the hearts of their followers and a grateful public.

I trust, young gentlemen, that at the beginning of your professional life, you will imitate the virtues and high attainments of your predecessors. Let Onward! be indeed your watchword in the career you have just begun. Progression now, more than ever before, is the inspiring theme in Medicine, and all the Arts and Sciences. Never lose an opportunity to add to your repertory of knowledge! You must search daily for medical truths and facts; investigate your subjects thoroughly, and in wisdom adapt your knowledge to the most practical purposes.

To explore the wonderful mysteries of the Medical world will require all the thought, application, energy, and enterprise you can possibly summon to your assistance, and to enable you to reach the goal of a noble ambition.

But do not be discouraged, when you survey the vast realm to be trodden by your untried feet. *Omnia vincit labor*, though so oft repeated, has not lost its grand significance and power. It is still the inspiring watchword to the worker in life's many fields of action; and you, as many have done, will find it "the golden spur to success"; yet, it is not so much the number of steps you take in your career that are to be considered, as the direction in which they are made, and the solidity of the ground beneath your feet.

Nature, reverently interpreted by Science, will furnish this solid ground. Nature is the Magna Matri of all science, and when the latter would come to her, it must be with the spirit and purpose of really being taught, before it can learn the story of her simple but grand mysteries.

I am glad to know that Science and Religion are now learning how to approach each other, and we are learning that the former is not independent of Religion; and both working together in harmony can rightly interpret Nature and reveal to man the marvellous wisdom and the wonderful love of God.

So, while I would have you strive for the highest attainments in your profession, my dear young friends, remember that "in the humblest business, as in the highest aims of life, success practically depends on the exercise of the moral quite as much as on that of the intellectual faculties." Knowledge by itself is only a small power. Character is to knowledge just what interest is to money. If you have a well-rounded and complete character, you will unite indissolubly the pure moral principles, the grand acquirements that your intellectual faculties may attain as a scholar, and a physician. Then your life will be like some sweet-toned instrument with all its strings in harmonious accord, and whose music will gladden and refresh those about you, in whatever community you may move. In building up this sublime status of character, a young man must choose with great caution his associates, and look closely to his moral and mental environments. He will seek the company of the pure, the wise, and good ; and, whether in private or public, seen and known of all men, he will look to it that every stone he adds to the structure is of granite solidity and without a flaw.

I am glad that an intelligent public, as well as many noble men of our profession, are awakening to the importance of purity of character and exalted principles in the Doctor of Medicine. The evidences of this are numerous and significant, and plainly point to the status of the physician and the profession that will be demanded by the people at no very distant day. I hope that you, my young professional brethren, will nobly strive to meet that demand. Mark out your course at once in that direction, and start upon it with a brave, determined spirit. Let no one intimidate or ridicule you into doing wrong, especially when your object is more to do good to others than to youiselves. The profession you have chosen is peculiarly one of service. A wise writer has said, "there is no dignity but of service," and that "God's nobility among men are those who do the most service."

To work nobly for a noble object, greatens and exalts a man's character. and, also, the work of his life. Such service can never be of a menial nature. It honors God, and He, in return, honors the man who, while striving to do good to others, finds that great good has come to himself, and that he has gained flattering recognition from his fellow men, which he has not sought nor expected.

"To live out the life of a true physicain with all its high purposes, is a peerless privilege, no matter at what cost of transient trouble or unremitting toil." Such a life holds out to the young physician the promise of great destinies. Meet them, my young friends, with courage and valor, with clean hands and a pure heart, and a determination to make them your own. In no other secular profession does a Christian gentleman stand so conspicuous as in yours ; and I beg you to strive for that exalted personality in your life and career.

All courageous persons who start out on any noble career, scarcely ever fail to "meet the lions on the way," but you will find them chained and powerless to harm, if you put your trust in Him who is Omnipotent, and who "never slumbers nor sleeps." He will lead you surely and safely, whether your feet are pierced with thorns, or tread upon the tender leaves of flowers. And when your days on earth, richly embroidered with the jewels of good deeds, draws to an honorable close, may you look up and see the heavens opened in the dazzling effulgence of immortal beauty, and hear a Voice sounding full and sweet down the radiant heights, "Come up higher, and receive thy well-earned and just reward—the crown of eternal life!"

The names of the graduating class are as follows:

J. B. Bearden..............................Georgia.
W. W. Burckhalter..........................Georgia.
L. L. Clement..............................Georgia.
W. M. Cole.................................Alabama.
W. D. Coursey..............................Georgia.
T. N. Courson..............................Georgia.
F. B. Fite.................................Georgia.
Dewell Gann................................Georgia.
W. P. Hall.................................Alabama.
W. L. Harper...............................Georgia.
J. A. Holcomb..............................Georgia.
J. T. Hunter...............................Georgia.
T. C. McBrayer.............................North Carolina.
A. S. Miller...............................Texas.
D. C. Montgomery...........................Georgia.
T. J. Moss.................................Georgia.
J. P. Motley...............................Alabama.
R. L. Neal.................................Georgia.
F. D. Nichols..............................Georgia.
W. W. Payne................................Mississippi.
M. W. Pharr................................Georgia.
G. B. Pledger..............................Georgia.
S. N. Pope.................................Georgia.
J. F. Powell...............................Georgia.
Felix Price................................Georgia.
S. P. Rampley..............................Georgia.
C. F. Riden................................Georgia.
J. E. Robbins..............................Alabama.
C. M. Scogin...............................Texas.
W. H. Thurmond.............................Georgia.

A. B. Tucker............................Georgia.
J. D. Walker............................Georgia.
L. V. White............................ Alabama.
A. M. Pattison..........................Georgia.

After the conferring of the degrees came the address of W. W. Burckhalter, the valedictorian appointed by the Class. The address was an unusually good one, and well delivered.

The annual oration was next delivered by Rev. J. B. Hawthorne, M. D. The address required fifty minutes in its delivery, and was a characteristic effort of this great orator, who is noted for his ability and eloquence. The remarks of Dr. Hawthorne were practical and instructive, and couched in strong, emphatic terms, designed to impress the young men in the principles which should govern them through life as physicians and as good citizens. Among the many terse, pointed and characteristic sentences of the orator, he stated that " whatever is worthy of being done at all is worthy of being done well," and, in order to make a deep and lasting impression, he said, " I had rather be a good tailor than a poor lawyer ; I would rather be a good baker than a poor actor, especially a female actor in the costume of her paramour ; I would rather be a success as an organ pumper than a failure as an organ player ; I would rather distinguish myself as a boot-black than to be laughed at as a painter ; I would rather succeed at pulling up stumps than to fail at pulling out teeth ; I would rather be an intelligent cobler than an ignorant doctor ; I would rather adorn a scavenger's cart than disgrace a professor's chair ; I would rather be a big peanut peddler than a small politician. God only knows what I had rather be than one of those sensational newspaper scavengers !" These and many other like utterances of the doctor were original, and spoken, as we doubt not, with the sincere desire to do good, and to stimulate the young graduates to aim high, and to take no second-rate stand in their profession. Such sentiments are eminently appropriate, and are worthy of being inscribed, as a perpetual reminder, upon the tablet of memory.

The presentation of the Prizes next followed. This pleasing duty was performed by Professor Nicolson, the Dean, upon the following persons, to wit :

The first honor--a gold medal, based upon the highest aggregate vote in all the branches—was conferred upon F. B. Fite, of Cartersville, Ga.

The second honor—a gold medal—for the next highest vote, was awarded to J. B. Bearden, Ellijay, Ga.

The Prizes for the best examination in the separate branches were awarded as follows:

In Surgery, by Professor Gaston—a Case of Instruments—to L. I. Clement, of Big Creek, Ga.

In Obstetrics, by Professor Powell—a gold medal—to G. B. Pledger, of Atlanta, Ga.

In Physiology, by Professor Word—a Drug Case—to L. V. White of Attalla, Ala.

In Practice, by Professor Bizzell—a gold medal—to W. W. Burckhalter, of Lexington, Ga.

A second prize by Professor Bizzell, to S. P. Rampley, of Georgia.

In Materia Medica, by Professor Roy—a Clinical Case—to C. F. Riden, of Cumming, Ga.

In Anatomy, by Professor Nicolson—a Case of Instruments—to W. D. Coursey, of Sterling, Ga.

In Diseases of the Eye, Ear, and Throat, by Professor Hobbs—an Opthalmoscope—to R. L. Neal, of Bold Springs, Ga.

In Chemistry, by Professor Burns—a Test Apparatus—to F. D. Nichols, Atlanta, Ga.

In Pharmacy, by Mr. Jo. Jacobs—a Percolater, etc.—to C. M. Scogin, of Texas.

In the intervals to the several parts of the programme, the audience were entertained by the most delightful music, which, with the floral offerings presented by the young ladies, and the congratulations which marked the close of the exercises, gave zest and animation to the occasion, which, indeed, was among the most pleasing and interesting of the kind that ever occurred in Atlanta.

